# Effect of TetR Family Transcriptional Regulator PccD on Phytosterol Metabolism of *Mycolicibacterium*

**DOI:** 10.3390/microorganisms12112349

**Published:** 2024-11-18

**Authors:** Peiyao Xiao, Delong Pan, Fuyi Li, Yuying Liu, Yang Huang, Xiuling Zhou, Yang Zhang

**Affiliations:** School of Pharmaceutical Sciences and Food Engineering, Liaocheng University, Liaocheng 252059, China; m19861908982@163.com (P.X.); 2210150302@stu.lcu.edu.cn (D.P.); 15653129517@163.com (F.L.); 18854045687@163.com (Y.L.); 18253054497@163.com (Y.H.)

**Keywords:** *Mycolicibacterium* sp. LZ2, propionyl-CoA, transcription factor PccD, metabolic regulation, androstenedione

## Abstract

Androstenedione (AD) is an important intermediate for the production of steroidal drugs. The process of transforming phytosterols into AD by *Mycolicibacterium* is mainly the degradation process of the phytosterol side chain, and the excessive accumulation of propionyl-CoA produced by *Mycobacterium* will produce toxic effects, which seriously restricts the transformation performance of strains. In this study, *Mycolicibacterium* sp. LZ2 (Msp) was used as the research object to study the transcription factor PccD of the TetR family, which has the role of propionyl-CoA metabolism regulation. By constructing overexpression and deletion strains of *pccD*, it was confirmed that *pccD* had an inhibitory effect on the transcription of propionyl-CoA carboxylase genes (*pccA* and *pccB*). Electrophoretic Mobility Shift Assay (EMSA) and DNase I footprint analysis demonstrated that PccD is directly involved in the transcriptional regulation of *pccA* and *pccB* and is a negative transcriptional regulator of the *pcc* operon. In the study of phytosterol transformation, the growth rate and bacterial viability of Msp-Δ*pccD* were higher than Msp, but the growth of Msp-*pccD* was inhibited. As a result of testing of intracellular propionyl-CoA levels and AD production yields, it was found that lower propionyl-CoA levels and higher AD production yields were observed in Msp-Δ*pccD*. The results expand the cognition of propionyl-CoA metabolism regulation and provide a theoretical basis and reference for the rational transformation of phytosterol transformation strains and secondary metabolite synthesis strains with propionyl-CoA as a substrate, which has important research significance.

## 1. Introduction

Steroids are the second largest class of drugs after antibiotics. It has remarkable physiological and pharmacological functions, such as anti-allergy, anti-infection and anti-shock [1,2]. Androst-4-ene-3, 17-dione (androstenedione, AD), the main steroid pharmaceutical intermediate, can synthesize almost all steroid hormone drugs [3]. The establishment of a technical system for the synthesis of AD by microbial transformation using phytosterols as substrates successfully solved the problem of depletion of natural resources for chemical synthesis using dioscin as raw material. This technology also opens up a new way for the sustainable development of the global steroid hormone industry and has become the main production method of steroid hormone drugs [4]. In recent years, with the rapid development of biological fermentation, enzyme transformation, and omics technology, *Mycolicibacterium* has been listed as an important production strain for the production of various steroid drug intermediates such as AD. On the basis of the strain, the sterol metabolic pathway was modified by means of genetic engineering breeding to produce a new modified strain. This method has qualitatively improved the production capacity of various steroidal drug intermediates such as AD. The process of *Mycolicibacterium* sp. LZ2 metabolizing phytosterol to synthesize AD is mainly the β-like oxidation process of the phytosterol side chain, which will produce a large amount of acetyl-CoA and propionyl-CoA, and the amount of propionyl-CoA is at least twice that of acetyl-CoA [5]. Existing studies have shown that propionyl-CoA inhibits enzymes of the central metabolic pathways, such as pyruvate dehydrogenase, succinyl-CoA synthetase, and citrate lyase [6,7,8,9]. Therefore, whether human or microbial, excessive accumulation of propionyl-CoA in cells will produce toxicity and a stress response [10,11,12,13].

In *Mycobacteriaceae* microorganisms, there are multiple pathways to metabolize propionyl-CoA for detoxification. One is the methyl citrate metabolism pathway (MCC) that converts propionyl-CoA to succinate [14]. The other is the methylmalonyl pathway (MMC) that converts propionyl-CoA to succinyl-CoA [15]. Propionyl-CoA carboxylase (PCC) plays a key role in the MMC pathway, so the MMC pathway is also called the PCC pathway [16]. In addition, methylmalonyl-CoA produced by propionyl-CoA will be further synthesized into polyketide virulence lipids, such as phthiocerol dimycocerosate (PDIM) and sulfolipid-1 [17]. Our previous study showed that intact MCC and MMC pathways also exist in *Mycolicibacterium* sp. LZ2, and interference with either pathway can affect AD synthesis [3,18]. Therefore, propionyl-CoA plays an important role in maintaining metabolic flux balance, energy supply, and detoxification during *Mycobacteriaceae* microorganisms’ utilization of phytosterol and odd-chain fatty acids [15,19,20]. However, for Msp to improve the yield and production efficiency of AD, it is necessary to enhance the degradation efficiency of the phytosterol side chain or increase the substrate concentration, which will lead to excessive accumulation of propionyl-CoA in cells [21,22]. Excessive accumulation of propionyl-CoA will lead to problems, such as a long conversion cycle and low product yield in AD production. Existing studies have shown that strengthening the MMC pathway in actinomycetes strains, such as *Saccharopolyspora erythraea* [23], *Actinosynnema pretiosum* [24], and *Streptomyces hygroscopicus* [25], can effectively increase the production of secondary metabolites. Our previous studies have demonstrated that overexpression of the β subunit gene (*pccB*) of PCC and co-expression with *ndh* in *Mycobacterium neoaurum* TCCC 11978 can achieve enhancement of the MMC pathway [3]. In *Actinomycetes,* acyl-CoA carboxylase (ACCase) is usually regulated by the TetR family of regulators. These regulators interact with various small molecule chemicals to regulate different physiological aspects of bacteria [26]. The TetR-type repressor (bkaR) was shown to regulate the branched-chain keto-acid metabolism in *M. smegmatis* and *M. tuberculosis*. A microarray analysis show that BkaR also has global regulatory functions, and some genes related to β oxidation can be directly regulated by it [27]. Therefore, in addition to regulating propionyl-CoA metabolism, BkaR may have the function of global regulation, and plays a regulatory role in metabolic processes such as phytosterol degradation, branched-chain keto acid metabolism and β oxidation. In *S. erythraea,* PccD, a homolog of BkaR, has a negative regulatory function on propionyl-CoA assimilation [16,28], in addition to the regulatory function of branched-chain amino acid degradation. Knockdown of its encoding gene *pccD* can relieve its inhibition of MMC and branched-chain amino acid degradation pathways, thus significantly increasing erythromycin yield [16]. Similarly, in *Streptomyces avermitilis*, a TetR Family Transcriptional Repressor (AccR) negatively regulates acetyl-CoA and propionyl-CoA production and carboxylation. Knockdown of the AccR-encoding gene increased abamectin production by 14.5% [29]. The TetR family transcriptional regulator (BkdR) in *Streptomyces albus* B4 chassis regulates the supply of precursors required for heterologous spinosad biosynthesis by controlling acetyl-CoA and propionyl-CoA assimilation. Deleting the BkdR-encoding gene increases the production of heterologous spinosad [26].

Transcription regulators play a regulatory role in the transcription level in response to environmental or cell signals. The changes in their expression and regulatory ability play an important role in maintaining the survival status and productivity of strains [30]. There are few reports on the metabolic regulation mechanism of propionyl-CoA, an intermediate product, in transforming phytosterols into AD by *Mycolicibacterium*. Our previous studies have confirmed that the transcription factor PrpR can regulate the level of propionyl coenzyme A in cells through the MCC pathway, thus affecting the synthesis of AD, but the molecular mechanism of the MMC pathway of propionyl coenzyme A metabolism in *Mycolicibacterium* sp. LZ2 has not been studied. In order to study the metabolism of propionyl coenzyme A by the MMC pathway and the molecular mechanism affecting the metabolism of propionyl coenzyme A, the research work of this paper was carried out. By consulting the literature, the transcription factor genes that have been identified to regulate the MMC pathway were compared with the whole genome of *Mycolicibacterium* sp. LZ2, and the target gene D174_19740 (*pccD*) with the highest similarity was located, encoding the TetR family regulatory protein (PccD), so the research on the regulation mechanism of transcription factor PccD was carried out. In order to study the effect of transcription factor PccD on the physiological properties of the strain, *pccD* knockout and overexpression strains were constructed to evaluate the growth status and AD production performance of the strain. In order to study the regulatory mechanism of transcription factor PccD on the adjacent *pccA* and *pccB* genes encoding propionyl-CoA carboxylase, RT-qPCR experiments were carried out. It was found that the transcription factor PccD had a negative regulatory effect on the *pccA* and *pccB* genes encoding propionyl-CoA carboxylase. In order to study whether the transcription factor PccD directly interacts with the regulatory regions of *pccA* and *pccB*, and to clarify the specific binding sites, EMSA and DNase I footprinting experiments were carried out. It was found that the transcription factor PccD binds to the upstream promoter region of *pccA* and *pccB* genes and inhibits propionyl coenzyme A metabolism. The molecular mechanism of the transcription factor PccD regulating target genes was clarified. The intracellular propionyl-CoA level was analyzed to elucidate the mechanism of transcription factor PccD’s influence on intracellular propionyl-CoA metabolism and the mechanism of transcription factor PccD’s influence on AD synthesis by regulating propionyl-CoA metabolism.

## 2. Materials and Methods

### 2.1. Strains and Cultivation Conditions

The strains, primers, and plasmids used in this study are shown in Table 1. *E. coli* DH5α was used to construct plasmids and cultured in a Luria–Bertani (LB) medium at 37 °C. The *Mycolicibacterium* sp. LZ2 (Msp) was used as the wild-type strain. Since Msp is highly homologous to *Mycolicibacterium neoaurum* VKM Ac-1815D, for which the complete genome sequence is available. pMV261 was used for gene overexpression; p2NIL and pGOAL19 were used to construct suicide knockout plasmids. The preparation of inclined colonies, seeds, and fermentation experiments were carried out according to the previous description [31].

### 2.2. Construction of Recombinant Strains

Gene overexpression and deletion methods have been reported in previous studies [18]. In Msp, the vector pMV261 with kanamycin (kan) resistance was used to overexpress the target gene. The *pccD* gene was amplified from the Msp genome and recombined with linearized pMV261 by the In-Fusion HD Cloning method to produce a recombinant plasmid designated pMV261-*pccD*. Electroporation introduced the recombinant plasmid into Msp, and the overexpression recombinant strain Msp-*pccD* was obtained. The steps of constructing the *pccD* knockout plasmid are described as follows: using the Msp genome as a template, PCR amplification obtained recombinant fragments of 1120 bp upstream and 1200 bp downstream of *pccD*. The two fragments were ligated to plasmid p2NIL, digested with PacI, and ligated to the selection marker cassette of pGOAL19 to construct the homologous recombination plasmid p2G19-*pccD*. The constructed plasmid was transferred into Msp cells by electroporation and screened for positive results, and the screened *pccD* knockout strain was named Msp-Δ*pccD*.

### 2.3. Isolation of RNA and Quantitative Reverse Transcription Polymerase Chain Reaction (qRT-PCR) Analyses

For qRT-PCR analysis, the strains were cultured for 60 h and collected by centrifugation at 8000× *g* for 10 min at 4 °C. RNA was extracted using the TRIzol Plus RNA Purification Kit (Invitrogen (Waltham, MA, USA), # 12183555) following the protocol provided with the kit, followed by cDNA synthesis using the FastKing RT Kit (TIANGEN (Beijing, China), # KR116). Real-time quantitative PCR was performed using SYBR Green PCR reagent (TIANGEN, # FP205). PCR cycle parameters: 95 °C for 15 min, then 40 cycles, 95 °C for 10 s, 55 °C for 30 s, 72 °C for 30 s, and finally extension at 72 °C for 10 min. The 16S rRNA was used as a control to normalize sampling errors [34]. Relative gene expression levels were calculated by the comparative Ct method (2^−ΔΔCt^ method) [35].

### 2.4. Protein Overexpression and Purification

The *pccD* gene was amplified from the Msp genome, and the fragment was ligated to the linear pET-30a (+) vector by a seamless cloning method. The recombinant plasmid was transferred into the competent state of *E. coli* BL21 (DE3), and the positive clone was detected and screened for sequencing verification. The successful recombinant plasmid was transferred into competent *E. coli* Rosetta (DE3) for expression. The monoclonal colonies were picked and cultured in LB liquid medium containing 30 µg/mL kanamycin and 34 µg/mL chloramphenicol at 37 °C for 12 h, and the bacteria were preserved. The plasmid was extracted for enzyme digestion verification. The successfully verified bacterial solution was inoculated into 50 mL LB liquid medium containing 30 µg/mL kanamycin and 34 µg/mL chloramphenicol for culture. When the OD value reached 0.6, 0.5 mM inducer IPTG was added and cultured at 20 °C overnight for a large amount of expression, and the cells were collected by centrifugation. The cells were dissolved with 50 mM Tris, 300 mM NaCl, 0.2 mM PMSF, 0.1% Triton X-100 (pH 8.0), sonicated, and the supernatant crude protein was collected by centrifugation. Take 5 mL of Ni-NTA and wash the equilibrium column with a binding buffer of 5 times the column bed volume. The crude protein was incubated with the equilibrated column packing for 1 h, and the effluent was collected. Clean the balancing column with a binding buffer. The column was washed with the washing buffer, and the effluent was collected. Elute with an elution buffer to collect the effluent. The crude protein and effluent components were treated separately, samples were prepared, and SDS-PAGE detection was carried out.

### 2.5. Gel Migration Experiment (EMSA)

PCR probe preparation was performed using puc57 plasmid with promoter sequences upstream of the *pccA* and *pccB* genes as templates and primers M13-f and M13-r with FAM fluorescent labels. The obtained PCR product was mixed with 6× loading Buffer and then detected by agarose gel electrophoresis with 2% TAE gel at 160 V for 15 min. All PCR products were recovered using the DiaSpin Column PCR Product Purification Kit (Sangon Biotech). The purified FAM-labeled DNA probe was combined with the purified gradient concentration of protein by reaction at room temperature 25 °C for 30 min using an EMSA kit (Sangon Biotech). Then, 6% non-denaturing PAGE gel was prepared, the EMSA reaction product was added to the gel wells, and the pre-electrophoresis solution containing 0.5× TBE non-denaturing gel was used for 60 V constant pressure for 30 min. Then electrophoresis at 170 V low temperature for 30 min, and the bands were detected by a fluorescent gel imager.

### 2.6. DNase I Footprinting

As in the EMSA reaction system, the purified FAM-labeled DNA probe was bound to the purified gradient protein concentration at room temperature at 25 °C for 30 min. After the reaction, add 5 µL of enzyme digestion system containing 10× React buffer (Thermo Fisher Scientific, Shanghai, China, EN0521), 1 M CaCl_2_, DNase I, and Nuclease-free water to the protein and DNA binding product, react at 37 °C for 55 s, then immediately add 10 µL of 0.5 M EDTA, mix well, and inactivate at 65 °C for 10 min to terminate the reaction. Then, the reaction product was recovered through the column with a DiaSpin column PCR product purification kit (Sangon Biotech) and sent to Shanghai Sangon Biotech for sequencing.

### 2.7. Determination and Analytical Methods

#### 2.7.1. Cell Growth and Bacterial Viability Detection

The growth of cells was determined by optical density in a medium free of phytosterols. However, cell growth in a culture medium containing phytosterols is challenging to measure by using this method. In this study, the cells extracted with ethyl acetate were resuspended in PBS, and then the absorbance value of OD_600_ was measured with a microplate reader to obtain the cell growth curve. Bacterial viability assays were performed using previously reported methods. The 2-(2-methoxy-4-nitrophenyl)-3-(4-nitrophenyl)-5-(2,4-disulfobenzene)-2H-tetrazole monosodium salt (WST-8) was reduced to formazan by bacteria, and its production amount and bacterial activity were positively correlated. After adjusting the OD_600_ value to 1 with pH 7.2 Tris-HCl buffer, 190 µL was added to a 96-well plate, and 10 µL of WST-8 were added to each well. After incubation at 30 °C for 1 h, the absorption value of 450 nm was detected with a microplate reader. Bacterial viability was reflected by the OD_450_ value.

#### 2.7.2. Determination of Intracellular Propionyl-CoA

Intracellular propionyl-CoA levels were detected using a modified method previously described by Xu et al. [16]. During the fermentation of Msp, Msp-*pccD* and Msp-Δ*pccD*, samples were taken every 24 h to analyze intracellular propionyl-CoA content. The collected cells were washed twice with pre-cooled phosphate-buffered saline (PBS, pH 8.0), then lysed on ice by adding lysis buffer (SDS lysate and 10% HCl) for 5 min, and shaken and milled for 2 min using a high-throughput tissue grinder (working for 10 s, stopping for 10 s), then frozen at −80 °C overnight, shaken and milled again for 1 min after thawing, and the supernatant was collected by centrifugation at 14,000× *g* for 10 min at 4 °C, and transferred to an equilibrated solid phase extraction column. After loading, the extraction column was washed with 95% methanol. This was followed by elution with 5% methanol. The collected eluate was stored at −80 °C until analysis. High performance liquid chromatography (HPLC) analysis was performed using a reversed phase C18 column (250 × 4.6 mm) at a column temperature of 25 °C. The two mobile phase solvents used were buffer A (methanol) and buffer B (100 mM ammonium acetate, pH 5.8), respectively. Linear gradient elution was performed at a flow rate of 0.5 mL/min with elution conditions: 10% to 20% A (0–5 min), 20% to 38% A (5–15 min), 38% A (15–17 min), 38% to 10% A (17–19 min), and data acquisition was stopped at 30 min. A calibration curve was prepared using a propionyl-CoA standard working solution. The propionyl-CoA concentration was calculated for each sample by inserting sample measurements into the standard curve.

#### 2.7.3. Analysis of Product AD

In the process of phytosterol conversion, samples were taken every 24 h, an equal volume of ethyl acetate was added to 0.8 mL of fermentation broth, ultrasonic extraction was carried out for 30 min, centrifugation at 12,000× *g* for 10 min, and 100 μL of the extract was dried at room temperature and dissolved in 1 mL of 80% methanol, centrifugation at 12,000× *g* for 20 min, and HPLC analysis was performed. HPLC analysis was performed according to the previously described method [31].

#### 2.7.4. Statistical Analysis Methods for Data in the Article

For the data that requires statistical analysis in the article, we used GraphPad Prism 8.02(263) and Origin 2024 for processing and conducted unpaired *t*-tests and ANOVA analysis. All data were repeated three times * *p* ≤ 0.05, ** *p* ≤ 0.01, *** *p* ≤ 0.001.

## 3. Results and Discussion

### 3.1. Genome Analysis Shows That the PCC Gene Operon Is Conserved in Actinomycetes

**Figure 1:**
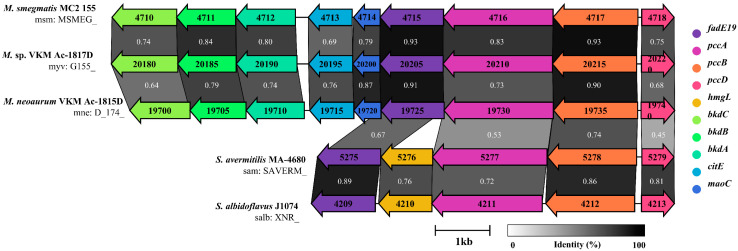
Genomic context of the PCC pathway gene cluster in mycobacteria and their close relatives. Grey shades represent conserved regions between genomes, and grey levels represent the Identity of adjacent genes, whose values are displayed in the shadows.

The PCC pathway-related gene cluster was analyzed for *Mycolicibacterium smegmatis* MC2 155 (msm), *Mycolicibacterium neoaurum* VKM Ac-1815D (mne), *Mycobacterium* sp. VKM Ac-1817D (myv), *Streptomyces avermitilis* MA-4680 (sma), and *Streptomyces albidoflavus* J1074 (salb) using the SSDB database of Kyoto Encyclopedia of Genes and Genomes (KEGG). Multiple putative *pcc* genes were present in all strains of interest, but the putative gene capable of constituting operon with the adjacent TetR-type repressor gene was unique. Through gene structure and sequence alignment analysis, the PCC pathway gene cluster was found to be conserved in actinomycetes (Figure 1). The regulator gene, which has been confirmed to have a regulatory effect on PCC, was compared with the whole genome gene of *M. neoaurum* VKM Ac-1815D, and the target gene with the highest similarity was located as D174_19740. The D174_19740 has 68% homology to *bkaR* (MSMEG_4718) of *M. smegmatis* MC2 155, 45% homology to *accR* (SAVERM_5279) of *S. avermitilis* MA-4680 (sma), and 43% homology to *bkdR* (XNR_4213) of *S. albidoflavus* J1074. By analyzing the genes adjacent to D174_19740, the results indicate that D174_19735 encodes the β subunit of propionyl-CoA carboxylase (PccB), and D174_19730 encodes the α subunit of propionyl-CoA carboxylase (PccA). The gene cluster of *pcc* in Msp was named *pcc* operon, and the genes homologous to D174_19730, D174_19735 and D174_19740 were named *pccA*, *pccB*, and *pccD*, respectively.

### 3.2. The Transcription Factor PccD Directly Inhibits the Expression of pccA and pccB Genes

**Figure 2:**
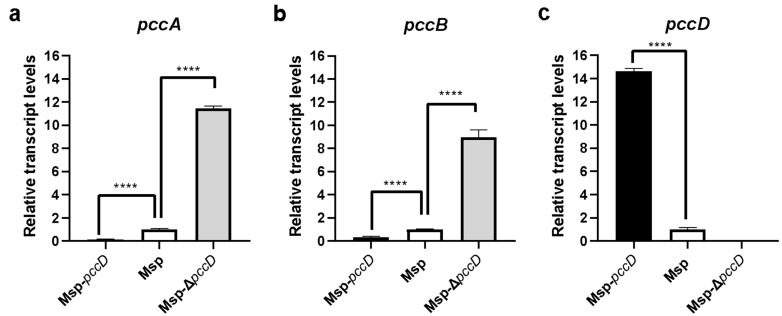
Gene expression levels of *pccA* (**a**), *pccB* (**b**), and *pccD* (**c**) in Msp, Msp-*pccD*, and Msp-Δ*pccD* detected by qRT-PCR. These values are the mean of the standard deviations of three replicate experiments. ****, *p* < 0.0001 (unpaired *t*-test).

To study the expression regulation of the transcription factor PccD on the adjacent *pccA* and *pccB*, a *pccD* overexpressing bacterium named Msp-*pccD* was constructed in Msp. A *pccD* knockout strain was constructed by homologous recombination and designated as Msp-Δ*pccD*. The strains were cultured to the logarithmic phase for 60 h, RNA was extracted, and the transcription levels of *pccA*, *pccB*, and *pccD* in Msp, Msp-*pccD*, and Msp-Δ*pccD* were analyzed by qRT-PCR (Figure 2). The results showed that after the *pccD* gene was knocked out, the transcription of *pccD* could not be detected. The transcription level of *pccD* in bacteria overexpressing Msp-*pccD* was 15 times higher than that of Msp. Correspondingly, deletion of the *pccD* gene resulted in an 11-fold and 9-fold up-regulation of the gene expression levels of *pccA* and *pccB*, respectively. By contrast, overexpression of *pccD* resulted in a 6-fold and 3-fold down-regulation of the gene transcription levels of *pccA* and *pccB*, respectively. These results suggest that the transcription factor PccD inhibits the expression of *pccA* and *pccB* genes.

### 3.3. Transcription Factor PccD Binds to Promoter Regions Upstream of pccA and pccB Genes

**Figure 3:**
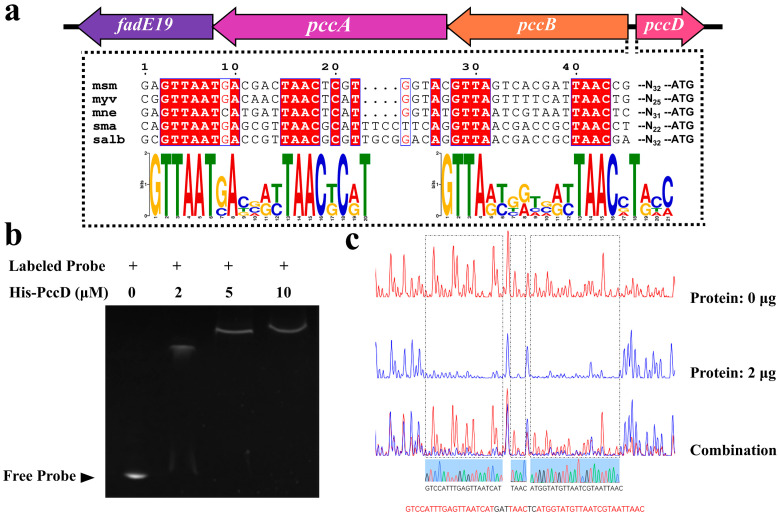
Transcription factor PccD binds to upstream promoter regions of *pccA* and *pccB* genes in Msp. (**a**) Genetic organization of the *pcc* operon in the Msp. (**b**) EMSA of His-PccD protein with upstream promoter regions of *pccA* and *pccB*. (**c**) Electropherograms of a DNase I digest of *pccA* and *pccB* promoter probe incubated with 2 μg of His-PccD.

The gene structure analysis of the *pcc* gene cluster showed that *pccA* and *pccB* share the same promoter, which is in the opposite direction to the downstream *pccD* gene. The intergenic regions between *pccA* and *pccB* and *pccD* are very short. Through sequence alignment, it is found that Msp has similar structures to the DNA binding sequences of BkaR and AccR of strains such as *M. smegmatis* MC2 155 and *S. avermitilis* MA-4680. Computational analysis of the *pccA* and *pccB* promoter of all the species tested using MEME identified a palindromic motif with two highly conserved cores (Figure 3a). Therefore, it is reasonable to assume that the TetR family protein PccD is a transcriptional regulator of *pccA* and *pccB*. To determine whether PccD directly interacts with the *pccA* and *pccB* regulatory regions, EMSA experiments were performed using FAM-labeled DNA probes of His-PccD and the upstream regions of the *pccA* and *pccB* genes. As shown in the results of in vitro experiments in Figure 3b, the motifs upstream of the *pccA* and *pccB* genes were significantly shifted in the bands after incubation with His-PccD protein (Figure 3b). Thus, the transcription factor PccD can bind to motifs upstream of the *pccA* and *pccB* genes. To elucidate the regulatory mechanism of PccD on the transcription of the *pccA* and *pccB* genes and determine the precise DNA binding site of PccD, DNase I footprinting experiments were performed with His-PccD protein and FAM-labeled probes made from the upstream regulatory regions of *pccA* and *pccB* genes. As can be seen from Figure 3c, there is apparent binding between the PccD protein and the probe of interest, forming a protected area on the probe. These results indicate that PccD specifically binds to the GTCCATTTGAGTTAATCAT and ATGGTATGTTAATCGTAATTAAC sequences within the *pccA* and *pccB* promoter regions. Thus, PccD inhibits transcription of the *pccA* and *pccB* genes by blocking the ligation of RNA polymerase to its promoter, thereby preventing transcription initiation and extension.

### 3.4. Regulation of Propionyl-CoA Metabolism by Transcription Factor PccD

**Figure 4:**
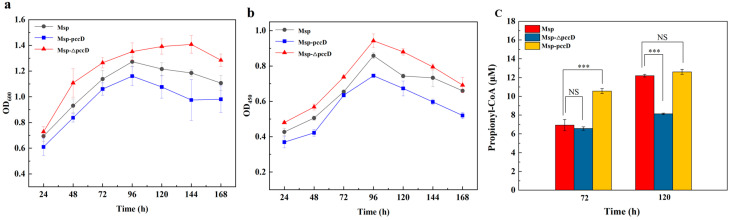
PccD negatively regulates the metabolism of propionyl-CoA. (**a**) Growth curves of strains Msp, Msp-*pccD* and Msp-Δ*pccD* on phytosterol medium. (**b**) Cell viability of strains Msp, Msp-*pccD* and Msp-Δ*pccD* on phytosterol medium. (**c**) Intracellular propionyl-CoA concentrations of strains Msp, Msp-*pccD*, and Msp-Δ*pccD* were cultured in a phytosterol medium for 72 h and 120 h. The error bars represent the standard deviation of the three biological replicates. NS *p* > 0.05, *** *p* ≤ 0.001. (ANOVA analysis).

Msp produces a toxic intermediate propionyl-CoA while metabolizing phytosterols to AD. Previous studies have shown that enhancing the intracellular PCC pathway is helpful to the growth of strains in a medium containing phytosterols, and the cell viability and AD transformation yield of recombinant bacteria are effectively improved [3]. To study the effect of PccD on the metabolism of phytosterols by Msp, Msp, Msp-*pccD*, and Msp-Δ*pccD* strains were cultured in a medium containing 5 g/L phytosterols, and samples were taken every 24 h to observe the growth and bacterial viability of each strain (Figure 4a,b). The results showed that the growth status and bacterial viability of Msp-Δ*pccD* were higher than those of Msp, which may be because the deletion of *pccD* relieved its inhibition of *pccB* and *pccA* gene transcription. The increase of PCC promoted the metabolism of propionyl-CoA. Contrary to the situation of the Msp-Δ*pccD* strain, the growth rate and bacterial viability of the Msp-*pccD* strain were lower than those of Msp. The reason may be that the excess PccD produced by overexpression of PccD enhances the inhibition of *pccB* and *pccA* gene transcription and thus reduces the production of PCC. Eventually, excess propionyl-CoA has a toxic effect on the strain. To verify this speculation, intracellular propionyl-CoA concentrations of Msp, Msp-*pccD*, and Msp-Δ*pccD* grown on a phytosterol-containing medium were measured. The results showed that the deletion of *pccD* did promote the metabolism of propionyl-CoA, resulting in the decrease of intracellular propionyl-CoA concentration, which was more obvious at 120 h than 72 h of fermentation; whereas overexpression of *pccD* led to an increase in intracellular propionyl-CoA concentration (Figure 4c). Taken together, these observations further demonstrate that PccD is a transcriptional repressor of the *pccB* and *pccA* genes, which attenuates propionyl-CoA metabolism by reducing PCC production.

### 3.5. Deletion of the pccD Gene in Msp Improves AD Yield

**Figure 5:**
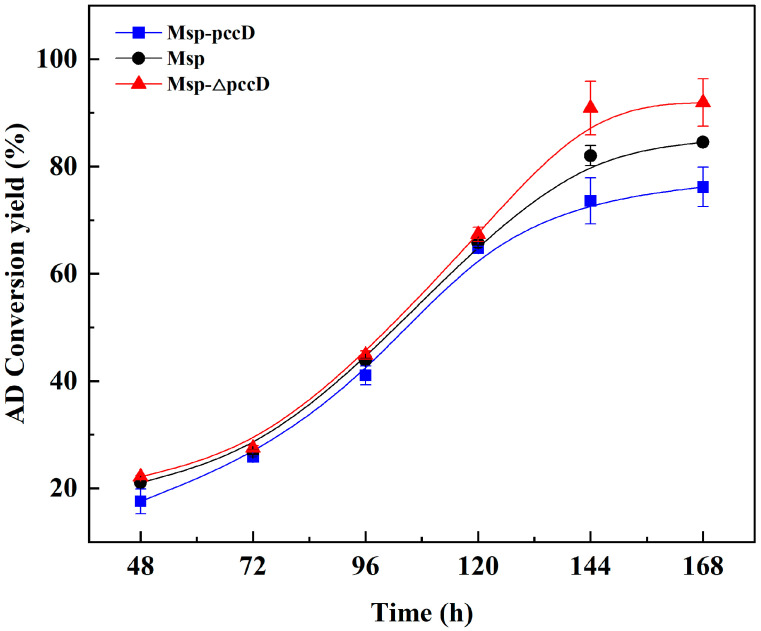
Yield of transformation of phytosterols into AD by strains Msp, Msp-*pccD* and Msp-Δ*pccD*. The error bars represent the standard deviation of the three biological replicates.

The process of transforming phytosterol into AD in *Mycolicibacterium* is mainly the process of phytosterol side-chain degradation. Excessive accumulation of propionyl-CoA produced by side-chain degradation will have toxic effects on strains and seriously restrict the performance of strains in transforming AD [2,18]. From the above results, it is speculated that the deletion of *pccD* can enhance propionyl-CoA metabolism by Msp, which is beneficial to the production of AD from phytosterols. To verify this speculation, the AD transformation yields of Msp, Msp-*pccD*, and Msp-Δ*pccD* strains were compared using a fermentation medium containing 5 g/L phytosterol. The results showed that the deletion of the *pccD* gene significantly improved the productivity of the strain. Compared with Msp, the Msp-Δ*pccD* strain increased the AD transformation yield by 8.75%. However, the AD transformation yield of the *pccD* overexpressing strain Msp-*pccD* decreased by 9.88% compared with the original Msp strain (Figure 5). It can be seen that PccD affects the AD production performance of strains by regulating the metabolism of propionyl-CoA.

## 4. Conclusions

The excessive propionyl-CoA produced by the conversion of phytosterols by *Mycolicibacterium* sp. LZ2 will have a toxic effect on the strain. The metabolism of propionyl-CoA needs to be strictly regulated to prevent its accumulation and reduce toxicity. There are two pathways metabolizing propionyl-CoA in *Mycolicibacterium* sp. LZ2, MCC, and MMC pathways. In the MMC pathway, propionyl-CoA generates succinyl-CoA through a series of enzymes such as propionyl-CoA carboxylase to further synthesize AD. However, because the molecular mechanism of the MMC pathway of propionyl-CoA metabolism in *Mycolicibacterium* sp. LZ2 is not clear, we carried out this study. In this study, the transcription factor genes identified in the literature that can regulate the MMC pathway were compared with the whole genome of *Mycolicibacterium* sp. LZ2, and the most similar target gene D174_19740 (*pccD*) was located, encoding the TetR family regulatory protein (PccD). Therefore, the regulation mechanism of transcription factor PccD was studied. EMSA and DNase I footprinting assays in vitro verified that the TetR family transcription factor PccD encoded by the *pccD* gene can directly interact with the upstream promoter region of the *pccB* and *pccA* genes encoding PCC on the MMC pathway to inhibit the transcription of the *pccB* and *pccA* genes.

**Figure 6:**
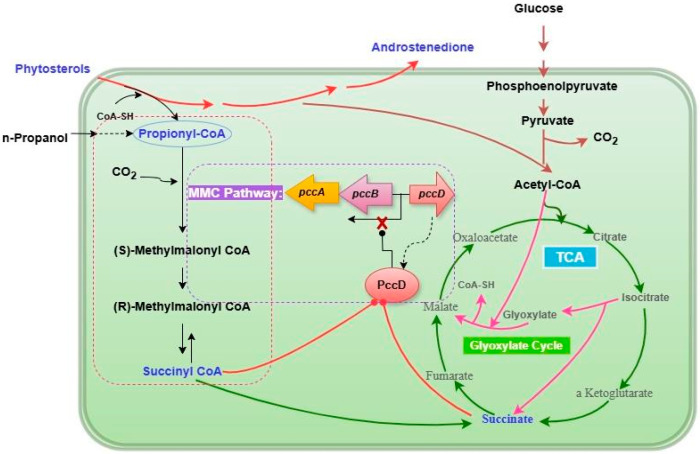
The regulatory mechanism model of transcription factor PccD regulating MMC pathway.

In this paper, succinate, an intermediate metabolite in the propionate metabolic pathway, was shown to affect the binding activity of the transcription factor PccD to the *pccB* and *pccA* genes. After the *pccD* gene was knocked down, the transcription of *pccD* could not be detected, while the transcription level of *pccD* in Msp-*pccD* strain was 15 times that of Msp. Correspondingly, deletion of the *pccD* gene resulted in an 11-fold and 9-fold increase in the gene expression levels of *pccB* and *pccA*, respectively; while overexpression of *pccD* resulted in a 6-fold and 3-fold decrease in the gene transcription levels of *pccB* and *pccA*, respectively. In addition, lower propionyl-CoA levels and higher AD conversion yields were observed in the Msp-Δ*pccD* strain compared to the wild-type strain. Based on the above experimental results, we proposed a regulatory mechanism model of the transcription factor PccD in *Mycolicibacterium* sp. LZ2 regulating the MMC pathway (Figure 6).

## Figures and Tables

**Table 1:** Plasmids, strains and primers used in this study.

Strains, Plasmids, and Primers	Significant Properties	Source or Purpose	References
strain			
Mutant of BNCC191574	Starting strain	BeNa Culture Collection (Xinyang, China)	[31]
*Escherichia coli* DH5α	General cloning host Transgen	Biotech (Beijing, China)	
*E. coli* BL21 (DE3)	Gene expression host	Sangon Biotech (Shanghai, China)	
Msp-pccD	pccD overexpressed This work strain of Msp	This work	
Msp-ΔpccD	Deletion of pccD in Msp	This work	
plasmid			
pMV261	Shuttle vector of Mycobacterium and Escherichia coli, carrying the heat shock hsp60 promoter, KanR	Overexpression of target genes	[32]
p2NIL	Plasmid for allelic exchange, non-replicative in Mycobacterium species, KanR	Knockout plasmid construction	[33]
pGOAL19	lacZ, hyg, and sacB marker genes cassette, HygR	Knockout plasmid construction	[33]
pMV261-pccD	pMV261 carrying an extra pccD for overexpression, KanR	This work	
p2G19-pccD	p2NIL carrying the homologous arms of pccD and the selection markers from pGOAL19	This work	
pET-30a (+)	*E. coli* expression plasmid	Sangon Biotech (Shanghai, China)	
pET-pccD	Insertion of pccD gene at pET-30a (+) multiple cloning sites for heterologous expression of PccD protein	This work	
Primer			
M13-f	GTTGTAAAACGACGGCCAG	PCR preparation of probes	
M13-r	CAGGAAACAGCTATGAC	PCR preparation of probes	
pccD-f	GGATCCAGCTGCAGAATTCATGCCCACTGAGACTCCGCG	pccD amplification	
pccD-r	CGCTAGTTAACTACGTCGACTCAGTGCGCGGCGGGTCAAGG	pccD amplification	
pccD-U-f	ATAAACTACCGCATTAAAGCTTACAACACGCCGTTGTTGGCG	pccD deletion	
pccD-U-r	CAGATCTCGATCGCCGGCCCGCTGACAC	pccD deletion	
pccD-D-f	CGGCGATCGAGATCTGGGTCGACGTACT	pccD deletion	
pccD-D-r	TGACACTATAGAATACATAGGATCCGTACATGACCACCGGCCGGG	pccD deletion	
16s-f-RT/16s-r-RT	GTAGGGTCCGAGCGTTGTC/GCGTCAGTTACTGCCCAGAG	Quantitative RT-PCR	
pccA-f-RT/pccA-r-RT	GATGGAACACGCGCTCAAAG/ACTCCTTGCTTGCGGTGATG	Quantitative RT-PCR	
pccB-f-RT/pccB-r-RT	TGTACGACGAATGTCCC/CTTCTTGACGGTGATCGGGT	Quantitative RT-PCR	
pccD-f-RT/pccD-r-RT	GCATTTCGCCAACAAGGAGG/TCGATGAGTCTGTCCAGTGC	Quantitative RT-PCR	

## Data Availability

The original contributions presented in the study are included in the article, further inquiries can be directed to the corresponding authors.
